# Effects of different remineralization agents on shear bond strength of orthodontic brackets: in vitro study

**DOI:** 10.1007/s00784-025-06312-6

**Published:** 2025-04-05

**Authors:** Gulsevim Oda, Ozge Muftuoglu Guler

**Affiliations:** 1 Department of Pedodontics, Faculty of Dentistry, Ankara Medipol University, Ankara, Turkey; 2 Department of Orthodontics, Faculty of Dentistry, Ankara Medipol University, Ankara, Turkey

**Keywords:** Orthodontic brackets, Shear bond strength, Remineralization agents, White spot lesions

## Abstract

**Objective:**

The aim of this study was to evaluate the effect of different remineralization agents-Proshield Fluoride Varnish (FV), GC MI Varnish (MI), and ROCS Medical Mineral Gel (ROCS MMG)-on the shear bond strength (SBS) of orthodontic brackets after application at various time intervals.

**Methods:**

A total of 120 human premolar teeth were divided into 10 groups (*n* = 12). The experimental groups received different remineralization agents (FV, MI, ROCS MMG) at time intervals of 1, 15, and 30 days, while the control group received no application. The enamel surfaces were etched, and metal orthodontic brackets were bonded using a light-cured composite adhesive. SBS was measured using a universal testing machine, and adhesive remnant index (ARI) scores were assessed after bracket debonding.

**Results:**

No statistically significant differences were observed in SBS among all experimental groups and the control group (*P* = 0.307). SBS values for the FV, MI, and ROCS MMG groups did not differ significantly at the 1, 15, or 30-day intervals. ARI scores also showed no significant differences between groups (*P* = 0.370).

**Conclusion:**

The application of Proshield Fluoride Varnish, GC MI Varnish, and ROCS Medical Mineral Gel at different time intervals did not affect the shear bond strength of orthodontic brackets. These remineralization agents can be safely used to prevent white spot lesions and caries before orthodontic treatment at time intervals of 1, 15, and 30 days without compromising bond strength.

**Clinical relevance:**

Proshield FV, GC MI Varnish, and ROCS MMG safely prevent white spot lesions and caries without affecting bracket bond strength.

## Introduction

The presence of orthodontic brackets in the mouth may makes mechanical removal of plaque difficult [1, 2]. In addition to increased biofilm accumulation around fixed orthodontic appliances, cleaning teeth by the tongue, cheeks, and lips during chewing also becomes more difficult [3, 4]. When insufficient oral hygiene is also considered, the susceptibility to white spot lesions may increase in patients undergoing fixed orthodontic treatment [5].

Clinically opaque, whitish, and chalky-appearance lesions, which are the first sign of mineral loss on the enamel surface, are termed White Spot Lesions (WSL). The development of WSL is increased by factors such as bacterial plaque accumulation, poor oral hygiene, frequent carbohydrate consumption and orthodontic appliances. Factors that reduce the risk of WSL include fluoride applications, mechanical plaque control, mouthwashes, casein phosphopeptides-amorphous calcium phosphate (CPP, ACP) containing products and adequate salivary flow. Saliva is critical for oral health and factors such as salivary flow rate, pH level and buffering capacity play an important role in determining the risk of WSL. Salivary pH usually ranges between 6.2 and 7.6, with a pH level of 6.7 considered normal. Saliva helps prevent WSL by maintaining the pH of the oral cavity at a neutral level [6–8].

To prevent decalcifications and white spot lesions that may occur during orthodontic treatment, it is important to maintain good oral hygiene, including proper brushing with fluoride toothpaste [9]. It has been reported that significantly more WSL develop in patients undergoing orthodontic treatment compared to those not receiving such treatment, and these lesions can cause aesthetic problems years after treatment [10]. Clinically, various therapies and agents have been used both before and during orthodontic treatment to reduce demineralization that may develop on the enamel surface. These include topical fluorides (high-fluoride toothpaste, fluoride mouthwashes, gels, and varnishes), application of CPP, ACP, mineral gel systems, antiseptics, probiotics, polyols, sealants, laser, resin infiltration, and micro abrasion [11, 12]. Pretreatment procedures are used to ensure remineralization before bracketing the teeth may interfere with the bonding strength of brackets [13].

Due to the high prevalence of WSL during orthodontic treatment, it is necessary to use appropriate remineralization agents on both demineralized and intact enamel to prevent these lesions while minimizing any negative impact on the shear bond strength (SBS) of brackets [14].

The ROCS Medical Mineral Gel System (ROCS MMG) is a new agent formulated with calcium, phosphate, and magnesium, designed to promote the remineralization of dental hard tissues. It functions by restoring mineral saturation and enhancing the resistance of tooth enamel [15]. Magnesium increases the mechanical strength of tooth enamel and thus contributes to the remineralization process. It slows down crystal growth, resulting in a more regular and harder structure. In addition, as the magnesium concentration increases, the hardness of the enamel surface improves. Due to these properties, magnesium stands out as an important component in enamel repair and in the development of remineralization materials [16, 17].

The application of a mineral complex gel containing magnesium and calcium demonstrated a positive remineralization effect on early-stage carious lesions as well as on non-carious enamel lesions [18]. It has also been reported that it provides successful results in preventing demineralization of the enamel around orthodontic brackets [19].

The impact of remineralizing agents on the bond strength of orthodontic brackets remains controversial. Various studies have investigated the use of different remineralization agents prior to fixed orthodontic treatment. However, variations in study protocols and methodologies pose challenges to the evaluation and comparison of their findings [13, 20].

To the best of our knowledge, no study has assessed the impact of ROCS MMG on the bond strength of orthodontic brackets or compared it to other agents. This study aims to evaluate the effect of remineralizing agents on shear bond strength and to determine the optimal timing between the application of these agents and the bonding procedures.

## Materials and methods

Ethical approval was obtained from the Ankara Medipol University Health Sciences Non-Interventional Research Ethics Committee (No: 2023/149).

Power analysis was conducted using G*Power Version 3.1.9.7 (Heinrich-Heine-Universität Düsseldorf, Düsseldorf, Germany) to determine the required sample size [21]. The analysis was based on a significance level of 0.05, a power of 0.80, and a minimum effect size of 0.633. The calculation indicated that at least ten specimens were needed per group [20].

### Sample Preparation

Human premolar teeth were selected in this study to ensure the most accurate results. A total of 120 freshly extracted human premolar teeth, collected over a 3-month period for orthodontic purposes, were used. The inclusion criteria required teeth to be free of caries, volumetric or structural anomalies and defects, restorations, and traumatic lesions. During collection, the teeth were cleaned of blood and soft tissue remnants and stored in distilled water. Prior to the application of remineralizing agents, each tooth’s enamel surface was scaled and polished with a rubber cup and fluoride-free pumice paste for 10 s using a low-speed handpiece. The teeth were subsequently stored in distilled water, which was changed daily, at room temperature (27 °C). Subsequently, the teeth were randomly divided into 10 equal groups (*n* = 12).

Groups were assigned as follows for the remineralization agents: Proshield Fluoride Varnish, GC MI Varnish, and ROCS MMG (Table 1):

**Table 1:** Composition of the remineralization agents

Material / Product	Composition
**Proshield Fluoride Varnish** (President Dental, Germany)	*Resin*,* Ethanol*,* Sodium Fluoride (5%)*,* Tricalcium Phosphate (TCP)*,* Xylitol (sweetener)*,* Flavoring agents*
**Medical Minerals Gel** (R.O.C.S. Medical Minerals; DRC-Group, Russia)	*Aqua*,* Glycerin*,* Xylitol*,* Hydroxyethylcellulose*,* Calcium Glycerophosphate*,* Polysorbate-20*,* Methylparaben*,* Magnesium Chloride*,* Hydroxypropyl Guar*,* Flavoring agents*
**MI Varnish** (GC, Tokyo, Japan)	*Sodium Fluoride*,* Casein Phosphopeptide-Amorphous Calcium Phosphate (CPP-ACP) (5%)*,* Polyvinyl Acetate*,* Hydrogenated Rosin*,* Ethanol*,* Silicon Dioxide*,* Strawberry flavor*

1. Control Group: No intervention (*n* = 12).

- Fluoride Varnish Group (*n* = 36).

(2) 1 day after Fluoride Varnish Application (*n* = 12) referred to as ***F1***.
(3) 15 days after Fluoride Varnish Application (*n* = 12) referred to as ***F15***.
(4) 30 days after Fluoride Varnish Application (*n* = 12) referred to as ***F30***.

- MI Varnish Group (*n* = 36).

(5) 1 day after MI Varnish Application (*n* = 12) referred to as ***MI1***.
(6) 15 days after MI Varnish Application (*n* = 12) referred to as ***MI15***.
(7) 30 days after MI Varnish Application (*n* = 12) referred to as ***MI30***.

- ROCS MMG Group (*n* = 36).

(8) 1 day after the ROCS MMG Varnish Application (*n* = 12) referred to as ***MMG1***.
(9) 15 days after ROCS MMG Varnish Application (*n* = 12) referred to as ***MMG15***.
(10) 30 days after ROCS MMG Varnish Application (*n* = 12) referred to as ***MMG30***.

### Application of remineralization agents

Each remineralization agent was applied according to the manufacturer’s instructions. In the Fluoride Varnish group (F1, F15, F30), the buccal surfaces of each tooth were partially dried, and Proshield Fluoride Varnish was applied using a micro brush. After 5 min, the varnish was rinsed off with distilled water. This process was repeated after 6 h for a total of 5 days [20].

The same application protocol was followed for the MI Varnish (MI1, MI15, MI30) and ROCS Medical Mineral Gel (MMG1, MMG15, MMG30) groups. The teeth were stored at room temperature in distilled water, which was changed daily, until the shear bond strength tests.

Brackets were bonded at different intervals post-application: 1 day after for groups F1, MI1, and MMG1; 15 days after for groups F15, MI15, and MMG15; and 30 days after for groups F30, MI30, and MMG30.

### Bracket bonding procedure

Prior to bonding the brackets, the buccal surfaces of the teeth were cleaned with a fluoride-free pumice paste using a prophy brush. The enamel was etched with 37% phosphoric acid for 30 s, then rinsed with water spray for 15 s and air-dried. Metal premolar brackets (Omniarch; Dentsply GAC, Bohemia, NY) were bonded using the Transbond XT light-cured composite adhesive system (3 M Unitek, Monrovia, CA). A uniform adhesive thickness was achieved by applying a force of 300 g to each bracket with a tension gauge (Correx Dentaurum, Ispringen, Germany) prior to light curing. The adhesive was cured using the Ultradent Valo Ortho Cordless LED curing light (Ultradent Products, South Jordan, UT). This device has a power output of 3200 mW/cm², and the adhesive was cured for 2 × 3 s from both the mesial and distal aspects of the brackets, as recommended by the manufacturer.

### Sample embedding and shear bond strength testing

**Fig. 1:**
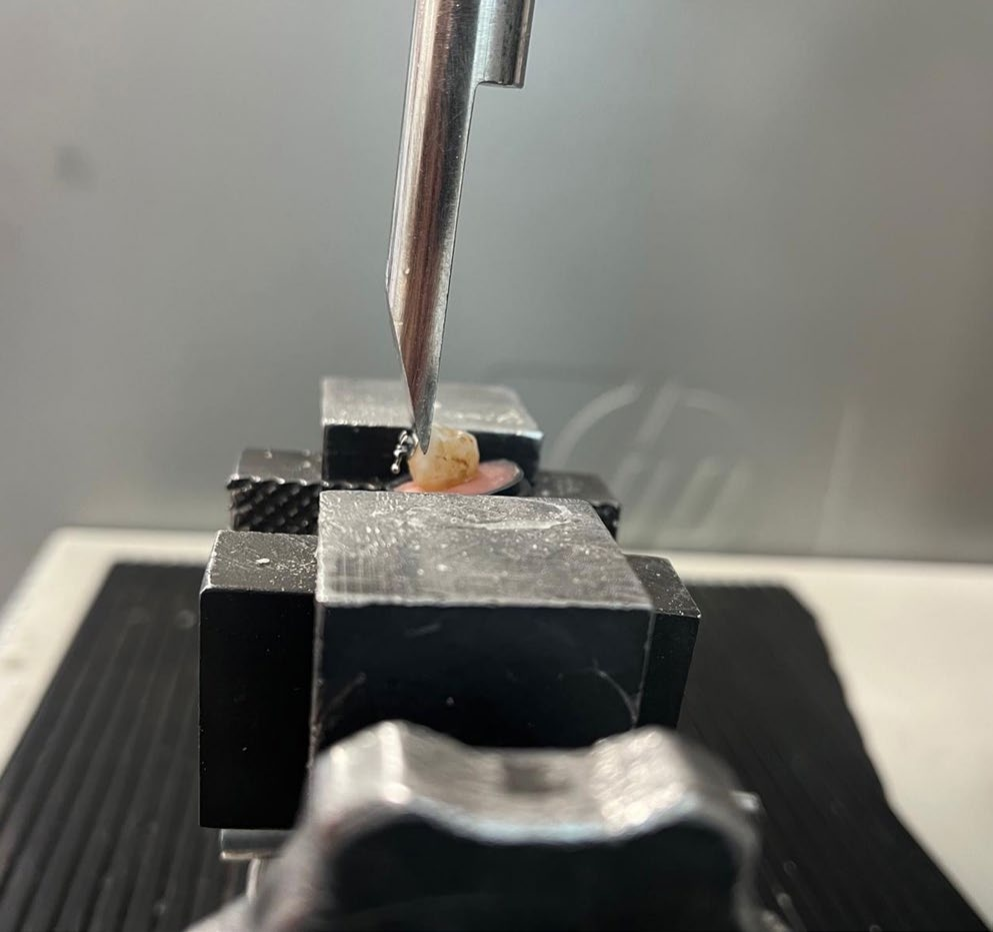
Universal testing device used in the study

Teeth were embedded in self-cured acrylic resin blocks using a mounting jig to ensure that the buccal surfaces were aligned parallel to the direction of the applied shear force. Shear bond strength testing was conducted with a universal testing machine (Instron, model 4204; Canton, MA, USA) that applied force in the occlusogingival direction at a crosshead speed of 1 mm/min, directly impacting the bracket base (Fig. 1). The force recorded in Newtons (N) was converted to megapascals (MPa) using the equation: MPa = debonding force (N) / bracket base area (12 mm²), where 1 MPa is equivalent to 1 N/mm² [1, 22].

Following bracket debonding, the adhesive remnants on the enamel surface were analyzed using a stereomicroscope (Carl Zeiss Inc., Oakland, CA, USA) at ×10 magnification. The adhesive remnant index (ARI) was utilized for assessment [23].

The ARI scores range from 5 to 1, where: 5 indicates no adhesive remains on the enamel surface, 4 means less than 10% of the adhesive is left, 3 corresponds to more than 10% but less than 90% of the adhesive remaining, 2 represents over 90% of the adhesive still adhering to the enamel, and 1 signifies that all of the adhesive remains on the enamel, displaying the outline of the bracket base.

### Statistical analysis

Statistical analysis was carried out using SigmaPlot 12.5 software (Systat Software Inc, San Jose, CA, USA). The Shapiro-Wilk test was used to assess the normality of the data distribution. For normally distributed (parametric) data, a one-way ANOVA was used, followed by Tukey’s post-hoc test to identify group differences. For nonparametric data, the Kruskal-Wallis test was used, with the Bonferroni (Dunn) method applied for post-hoc comparisons. The chi-square test was used to evaluate if there were significant differences in the ARI scores between the groups. A p-value of less than 0.05 was considered statistically significant.

## Results

No statistically significant differences were observed when analyzing the shear bond strength (SBS) scores among all groups (*P* = 0.307) (Table 2).

**Table 2:** Descriptive statistics of the groups and comparison of SBS (MPa) values

Group	*n*	Mean	SD	Min	Max	Median	*P* value
Control	12	23,92	9,8	7,06	36,65	25,49	*P* = 0,307^a*^ *P* = 0,264^b^ *P* = 0,712^c*^ *P* = 0,605^d*^ *P* = 0.106^e^ *P* = 0,103^f*^ *P* = 0.242^g^ *P* = 0.356^i^ *P* = 0.931^j*^ *P* = 0.983^k*^
F1	12	18,89	5,12	9,75	24,86	20,33	
F15	12	17,59	5,89	9,59	16,71	18,48	
F30	12	13,88	4,66	6,77	18,03	15,90	
MI1	12	13,98	5,51	8,25	22,36	12,89	
MI15	12	17,13	6,07	8,67	23,62	17,19	
MI30	12	16,25	4,9	10,38	22,58	16,57	
MMG1	12	18,32	7,7	7,9	31,24	17,56	
MMG 15	12	16,12	8,33	6,45	30,95	15,61	
MMG 30	12	14,86	5,53	4,79	20,47	15	

* Kruskal Wallis, ^a^ Comparison between all groups, ^b^ Comparison between F groups, ^c^ Comparison between MMG groups, ^d^ Comparison between MI groups, ^e^ Comparison between FV groups and control group, f Comparison between MI groups and control group, ^g^ Comparison between ROCS MMG groups and control group, ^i^ Comparison between FV1-MI1-MMG1 groups, ^j^ Comparison between FV15-MI15-MMG15 groups, ^k^ Comparison between FV30-MI30-MMG30 groups

No significant differences were observed within the FV groups (*P* = 0.264), the MI groups (*P* = 0.605), and the ROCS MMG groups (*P* = 0.712). Comparisons between each experimental group and the control group also did not show any statistically significant differences: FV groups and control (*P* = 0.106), MI groups and control (*P* = 0.103), ROCS MMG groups and control (*P* = 0.242).

Furthermore, comparisons of the remineralization agents at different time intervals showed no significant differences between FV1-MI1-MMG1 (*P* = 0.356), between FV15-MI15-MMG15 (*P* = 0.931), and between FV30-MI30-MMG30 (*P* = 0.983). Lastly, assessment of the ARI scores among the groups also revealed no significant differences (*P* = 0.370) (Table 3).

**Table 3:** Frequency of ARI scores

Group	*n*	ARI = 1 (*n*)	ARI = 2 (*n*)	ARI = 3 (*n*)	ARI = 4 (*n*)	ARI = 5 (*n*)	*P* value
Control	12	0	0	4	4	4	0.370
F1	12	0	0	0	4	8	
F15	12	0	0	0	6	6	
F30	12	0	0	4	4	4	
MI1	12	0	0	1	5	6	
MI15	12	0	0	0	6	6	
MI30	12	0	0	6	4	2	
MMG1	12	0	0	0	10	2	
MMG 15	12	0	0	2	6	4	
MMG 30	12	0	0	0	8	4	

## Discussion

Caries development during orthodontic treatment affects the process of orthodontic treatment. Although remineralization agents are used to prevent caries development during orthodontic treatment, their effect on the SBS of orthodontic brackets is still controversial. In this study, the effects of Proshield Fluoride Varnish and GC MI Varnish, which are remineralization agents whose effects have been previously studied in the literature, and ROCS Medical Mineral Gel, which has not yet been studied in the literature on bracket bonding, on the SBS of orthodontic brackets were evaluated. Daneshkazemi et al. found no differences in SBS between fluoride varnish, ICON, CPP-ACP, and control groups on intact enamel surfaces, which is consistent with our findings [14]. Kimura et al. and Park et al. reported no significant differences in SBS when comparing fluoride varnish and CPP-ACP applications to control groups [24, 25]. Similarly, Naseh et al. compared remineralization treatments on 60 premolar teeth, including CPP-ACP, fluoride mouthwash, and a combination of both, finding no significant differences among the groups [26]. These studies collectively suggest that the use of remineralization agents does not compromise the bonding strength of orthodontic brackets similar to our study.

However, the literature also presents conflicting results, which may be attributed to variations in study design, application protocols, and the type of teeth used. For example, Kecik et al. reported higher SBS values in experimental groups treated with APF and CPP-ACP compared to a control group [27]. But their study utilized bovine teeth, which may exhibit different bonding characteristics compared to human teeth. In contrast, our study, which used human premolars, found no significant differences in SBS, highlighting the importance of considering the type of teeth when interpreting results. Additionally, Cossellu et al. observed that fluoride varnish resulted in the lowest SBS values, while Domantaitė and Trakinienė reported reduced SBS when brackets were placed one day after fluoride varnish application [13, 20]. These discrepancies may be due to differences in surface preparation methods, such as the absence of pumice paste in some studies, which could leave residual fluoride on the enamel surface and interfere with bonding. In our study, meticulous surface preparation, including the use of pumice paste and daily distilled water changes, likely minimized the residual effects of remineralization agents, contributing to the consistent SBS values observed across all groups.

The adhesive remnant index (ARI) scores in our study also showed no significant differences among the groups, which is consistent with findings from Cacciafesta et al. and Tabrizi and Cakirer [28, 29]. This further supports the conclusion that the application of remineralization agents does not adversely affect the SBS.

In our study, it can be considered that the acid etching process following the application of remineralization agents did not completely affect the remineralized enamel surface. This suggests that the mineral deposits formed by the remineralization agents on the enamel surface may have been partially resistant to the acid etching process. As a result, although the effect of remineralization agents on the SBS was not statistically significant, it caused a slight decrease. This slight decrease may be attributed to the structural changes induced by the remineralization agents on the enamel surface. However, it can be concluded that this effect is clinically insignificant and does not adversely affect the bracket bonding process.

While the primary focus of this study was on SBS, it is worth noting that ROCS MMG, a relatively understudied remineralization agent, has been reported in the literature to exhibit superior remineralization properties. For instance, Lale et al. demonstrated that ROCS MMG, when used with fluoride-free toothpaste, was more effective in preventing enamel demineralization around orthodontic brackets compared to fluoride toothpaste and fluoride varnish [19]. Similarly, Damar et al. found that ROCS MMG significantly increased enamel microhardness, whereas CPP-ACP did not produce statistically significant results [30].

Although our study did not evaluate the remineralization efficacy of ROCS MMG, its use as a prophylactic agent before bonding appears to be safe and effective, as it did not reduce SBS. This suggests that ROCS MMG could be a valuable addition to orthodontics for preventing decalcification and white spot lesions during treatment.

The absence of significant differences in SBS when remineralization agents were applied at different time intervals (1, 15, and 30 days) further underscores the stability of bonding outcomes over time. This finding is particularly relevant for clinical practice, as it indicates that the timing of remineralization agent application does not influence bracket bonding. The consistent results across all groups in our study may be attributed to the rigorous surface preparation protocol, which effectively removed any residual agents and ensured optimal enamel surface conditions for bonding.

Our study has some limitations. This study was conducted in vitro, and variables present in the oral environment, such as saliva, diet, and other biological factors, were not evaluated. Additionally, the shear bond strength was not assessed following aging regimens. Future studies should focus on more comprehensive clinical research to evaluate the long-term effects of remineralizing agents under conditions that closely replicate the oral environment, including factors such as saliva, aging of teeth, dietary habits and biological variations.

## Conclusion

In conclusion, this study demonstrates that the application of Proshield Fluoride Varnish, GC MI Varnish, and ROCS Medical Mineral Gel for 1, 15, and 30 days did not affect the SBS of orthodontic brackets. These results suggest that these remineralization agents can be used to prevent white spot lesions and caries that may occur before orthodontic treatment, without negatively affecting the SBS of orthodontic brackets. Further research under clinical conditions is necessary to confirm these findings.

## Data Availability

No datasets were generated or analysed during the current study.
